# Combining diagnostic memory clinic with rehabilitation follow-up after hip fracture

**DOI:** 10.1007/s41999-020-00334-x

**Published:** 2020-05-26

**Authors:** Roope Jaatinen, Tiina Luukkaala, Matti Viitanen, Maria S. Nuotio

**Affiliations:** 1 Department of Geriatric Medicine, Seinäjoki Central Hospital, Hanneksenrinne 7, 60220 Seinäjoki, Finland; 2 Department of Geriatric Medicine, University of Turku, 20014 Turku, Finland; 3 Research, Development and Innovation Center, Tampere University Hospital, Teiskontie 35, 33521 Tampere, Finland; 4 Health Sciences, Faculty of Social Sciences, Tampere University, 33014 Tampere, Finland; 5 Department of Clinical Geriatrics, Karolinska Institutet, Karolinska University Hospital, Huddinge, 14186 Stockholm, Sweden; 6 Turku City Hospital, Kunnallissairaalantie 20, 20700 Turku, Finland; 7 Turku University Hospital and University of Turku, Kiinamyllynkatu 4-8, 20521 Turku, Finland; 8 Present Address: Ensonkuja 6b, 02140 Espoo, Finland

**Keywords:** Cognitive disorder, Physical functioning, Hip fracture, Older people

## Abstract

**Purpose:**

Cognitive impairment and dementia are common in older hip fracture patients. We describe new diagnoses of cognitive disorders (NDCDs) and associated factors in a two-year post-hip fracture follow-up including the use of the diagnostic facilities of a memory clinic.

**Methods:**

Data were collected on admission and at outpatient assessment 4–6 months post-hip fracture. Diagnoses of cognitive disorders followed the evidence-based Finnish national care guideline including internationally accepted criteria. NDCDs up to 2 years post-hip fracture were extracted manually from the patient files. Logistic regression models were computed to examine the associations between the pre-fracture factors and the domains of the outpatient geriatric assessment and NDCDs.

**Results:**

Of the 1165 hip fracture patients aged ≥ 65 years, 831 had no previous diagnosis of cognitive disorder. Of these, NDCD was documented in 23.3%. Alzheimer’s disease (AD) with or without vascular cognitive impairment (VCI) was the most common diagnosis. Cognitive disorder was usually at a moderate stage. Age, higher ASA score and poor nutritional status on admission were associated with new cognitive disorders. At the outpatient follow-up, poorer activities of daily living and mobility disability were associated with NDCD. Patients with a NDCD were more likely to suffer greater mobility impairment, poorer nutritional status and to have more supported living arrangements at follow-up than in the pre-fracture situation.

**Conclusion:**

NDCDs are common after hip fracture and associated with impaired rehabilitation outcomes and poor nutritional status. A post-hip fracture assessment co-organized in the form of a memory clinic seems to be feasible to detect previously undiagnosed cognitive disorders. Earlier diagnosis of cognitive disorders is warranted.

## Key summary points

**Aim:**

To specify the various diagnoses of cognitive disorders detected in post-hip fracture follow-up.

**Findings:**

Previously undiagnosed cognitive disorders are common in older hip fracture patients and are associated with impaired physical functioning and poor nutritional status.

**Message:**

The assessment of cognitive impairment is important alongside the comprehensive evaluation of hip fracture rehabilitation.

## Introduction

Cognitive disorders and falls are two significant issues of the globally ageing population [1]. The causal relationship between the two is considered multifactorial; cognitive disorders impair gait control, executive functions and stability, leading to increased risk of falls and related injuries [2, 3]. Up to 97% of hip fractures occur as a consequence of falls [4].

Cognitive impairment and dementia are common in hip fracture patients: studies have reported prevalences from 40% up to 85% [5]. Prefracture cognitive disorders have been found to be associated with adverse outcomes after hip fracture [6]. The definition of cognitive impairment frequently varies between studies [5, 7] and is regularly pooled under one term; *dementia*—an umbrella term for a range of neurodegenerative and vascular brain disorders. Normal and impaired cognitive capacity are often differentiated by accepted tools, such as the mini-mental state examination (MMSE), the Montreal cognitive assessment (MoCA), and clinical dementia rating (CDR) [8]. Studies are rarely more specific about diagnoses.

According to the evidence-based Finnish care guideline, cognitive disorders are diagnosed by a specialist in either geriatric medicine or neurology [9]. For this purpose, geriatric memory clinic services have been established nationwide. Reaching a diagnosis is important to identify treatable causes, to slow down a progressive condition, to prevent secondary risks, to provide proactive lifestyle guidance, and to allow patients and families adapt to the situation [10]. In patients with hip fractures, it is not feasible to carry out diagnostic investigations of cognitive disorders during the acute phase of hip fracture care because of the probable confounding effects, notably acute delirium and the effect of opioid medication on cognition.

We have developed a post-hip fracture pathway, where all patients are invited for a comprehensive geriatric assessment (CGA) at the geriatric outpatient clinic 4–6 months postoperatively [11, 12]. The assessment provides an opportunity to check rehabilitation outcomes and for secondary prevention of subsequent falls and fractures. Moreover, the assessment includes all the functions and features of a diagnostic memory clinic. We have previously reported that up to 56% of hip fracture patients with no prefracture diagnosis of a cognitive disorder scored less than 24 points on the MMSE carried out at the outpatient assessment, thereby suggesting the presence of a significant proportion of previously undiagnosed cognitive disorders [12].

This study aims to describe new diagnoses of cognitive disorders (NDCDs) identified during a two-year period post-hip fracture in a population based cohort of consecutive hip fracture patients. The follow-up included a visit to our geriatric outpatient clinic with facilities to diagnose cognitive disorders. In addition, we aim to describe the association of NDCDs in patients attending the postoperative outpatient assessment with prefracture factors and domains of the outpatient assessment with a focus on cognitive performance, physical functioning and nutritional status.

## Materials and methods

### Study population

All hip fracture patients aged ≥ 65 years from a population of approximately 200,000 in a geographically defined area undergo the same locally developed treatment pathway [13]. Patients sustaining their first hip fracture between January 2010 and August 2015 were included. Pathological and periprosthetic fractures were excluded. Patients with previously diagnosed cognitive disorder were excluded from the two-year follow-up. A multidisciplinary geriatric team (geriatric nurse, physiotherapists, and geriatrician) carried out data collection, which commenced on admission to hospital and continued with a clinical outpatient assessment to which all the patients were invited 4–6 months post-operatively. The cognitive disorders were specified according to clinical diagnoses following the evidence-based national care guideline valid at the time [9], which embodies currently accepted international criteria [14, 15]. The national care guidelines are regularly updated.

### Data collection

Known pre-fracture diagnosis of cognitive disorder (yes or no)—as diagnosed by a specialist in geriatric medicine or neurology—was elicited from patients, caregivers or nurses and confirmed from the electronic patient files.

Data on American Society of Anesthesiologists (ASA) score were registered and categorized to 1–2 (healthy patients or patients with mild systemic disease), 3 (patients with severe systemic disease), or 4–5 (patients with systemic disease as a constant threat to life or a morbid patient not expected to survive the operation). The geriatric nurse assessed the nutritional status with the short form of the mini nutritional assessment (MNA-SF). It was categorized to scores 12–14 (normal nutritional status), 8–11 (at risk of malnutrition), and 0–7 (malnourished). Number of medications in regular use, mobility and living arrangements were also documented at the time of the hip fracture together with age and gender. See Table 1 for details.

**Table 1:** Distributions of baseline variables at the time of the hip fracture

	No cognitive disorder, (*n* = 347)	Cognitive disorder, (*n* = 194)	*P*	Age- and sex-adjusted cognitive disorder, yes vs. no
				OR (95% CI)
Age, mean (SD)	80.1 (7.68)	83.7 (6.37)	< 0.001	1.08 (1.05–1.11)
Age n (%)	81 (IQR: 74–86)	85 (IQR: 80–88)	< 0.001	
Age			< 0.001	
65–79	156 (45.0)	46 (23.7)		1.00
80–90	154 (44.0)	116 (59.8)		2.68 (1.76–4.06)
> 90	37 (10.7)	32 (16.5)		3.13 (1.74–5.64)
Gender, *n* (%)			0.876	
Female	265 (76.4)	147 (75.8)		1.00
Male	82 (23.6)	47 (24.2)		1.31 (0.85–2.02)
Comorbidity, ASA score, *n* (%)			0.001	
1–2	93 (26.8)	28 (14.4)		1.00
3	213 (61.4)	124 (63.9)		1.59 (0.97–2.61)
4–5	38 (11.0)	39 (20.1)		2.61 (1.37–4.95)
Unknown	3 (0.9)	3 (1.5)		3.93 (0.72–21.32)
Nutritional status, MNA-SF, *n* (%)			0.001	
12–14	245 (70.6)	104 (53.6)		1.00
8–11	77 (22.2)	69 (35.6)		1.95 (1.30–3.78)
≤7	9 (2.6)	10 (5.2)		2.23 (0.86–5.33)
Unknown	16 (4.6)	11 (5.7)		1.50 (0.66–3.40)
Number of medications, *n* (%)			0.054	
< 4	103 (29.7)	49 (25.3)		1.00
5–10	205 (59.1)	110 (56.7)		1.07 (0.70-1.63)
> 10	39 (11.2)	35 (18.0)		1.85 (1.04–3.31)
Mobility, *n* (%)			< 0.001	
Independent	275 (79.3)	120 (61.9)		1.00
Non-independent	70 (20.2)	72 (37.1)		2.05 (1.36–3.07)
Unknown	2 (0.6)	2 (1.0)		2.06 (0.27–15.56)
Form of living arrangements, *n* (%)			< 0.001	
Own home with or without home care	312 (89.9)	155 (79.9)		1.00
Assisted care facility	30 (8.6)	39 (20.1)		2.39 (1.40–4.08)
Unknown	5 (1.4)	0 (0)		–

Age- and gender adjusted association of the variables with new diagnosis of cognitive disorder in a systematic two-year follow-up among those who attended the geriatric outpatient assessment (*n* = 541)

*SD* standard deviation, *IQR* interquartile range, *ASA* American Society of Anesthesiologists, *MNA-SF* mini nutritional status-short form, *OR* odds ratio, *CI* confidence interval

The follow-up visit was organized to include input from a multidisciplinary team. Both the patient and his or her next of kin or caregiver were invited. The diagnostic procedures were initiated if there was a clinical suspicion of a previously undiagnosed cognitive disorder either during acute hip fracture care or at the outpatient follow-up. The diagnostic protocol followed the 2010 update of the evidence-based national care guideline on memory disorder. Medical history was taken by interviewing the patient and the next of kin or caregiver separately. At least the MMSE, clock drawing test (CDT) and CDR were used to assess cognition. For some patients, the Consortium to Establish Registry for Alzheimer’s disease (CERAD) test [16] was completed before the outpatient visit by a local memory nurse. Neuropsychological examinations carried out by a trained psychologist were also available at the outpatient clinic for purposes of differential diagnostics.

MMSE was carried out by experienced geriatric nurses and patients were categorized into normal cognition > 25 and mild 21–25, moderate 12–20 or severe < 12 cognitive dysfunction [17]. CDT was used to assess visuo-constructive abilities, neglect and spatial dysfunction [18]. Severity of cognitive impairment was assessed according to CDR and categorized into three classes: no or possible dementia CDR 0-0.5, mild dementia CDR 1, moderate or severe dementia CDR 2–3 [17]. Basic and instrumental activities of daily living (BADL, IADL) were assessed according to Katz and Lawton–Brody, respectively [19, 20].

Basic laboratory tests were conducted to eliminate treatable causes of cognitive impairment such as hypothyreosis, hypercalcaemia and deficiencies in vitamin B12 or folic acid. Computerized tomography brain scan assessed by experts in neuroradiology was used as the imaging technique for diagnostic evaluation on each patient with suspected cognitive disorder [21]. Intracranial expansions were excluded and vascular lesions as well as local atrophies of the medial temporal lobe and hippocampal regions were scrutinized. The main categories of the cognitive disorders were Alzheimer’s disease (AD), vascular cognitive impairment (VCI), mixed cognitive impairment (AD + VCI), Lewy body dementia and Parkinson’s disease-related dementia. The diagnostic criteria for each type of cognitive disorder followed the currently valid care guideline on cognitive impairments [9, 14, 15]. Patients with mild cognitive impairment (MCI) were not included in the diagnoses. Finally, an individual care and rehabilitation plan was designed for each patient together with the multidisciplinary team. An experienced geriatrician or a resident in geriatrics under her supervision set the diagnoses of cognitive disorders.

A physiotherapist’s assessment preceded the geriatric assessment. The timed up and go-test (TUG) and elderly mobility scale (EMS) were documented (Table 2). The TUG test [22] was used both as time and as qualitatively assessed by the examining physiotherapists and categorized as shown in Table 2. EMS test was categorized according to the validated structures [23].

**Table 2:** Distribution of the domains of the post-hip fracture comprehensive geriatric assessment in relation with new diagnosis of cognitive disorder (*n* = 541)

	No cognitive disorder, (*n* = 347)	Cognitive disorder, (*n* = 194)	*P*	Age and sex adjusted cognitive disorder, yes vs. no
	*n* (%)	*n* (%)		OR (95% CI)
Cognition, MMSE				
26–30	139 (40.1)	9 (4.6)		1.00
21–25	119 (34.3)	46 (23.7)		5.56 (2.60–11.9)
12–20	42 (12.1)	96 (49.5)		32.2 (14.8–70.2)
< 12	5 (1.4)	18 (9.3)		53.2 (15.7–180)
Unknown	42 (12.1)	25 (12.9)		8.42 (3.57–19.8)
Cognition, CDT			< 0.001	
5–6	139 (40.1)	17 (8.8)		1.00
2–4	118 (34.0)	75 (38.7)		4.55 (2.52–8.23)
0–1	35 (10.1)	71 (36.6)		14.31 (7.41–27.6)
Unknown	55 (15.9)	31 (16.0)		3.58 (1.78–7.18)
Cognition, CDR			< 0.001	
0–0.5	127 (36.6)	10 (5.2)		1.00
1	92 (26.5)	46 (23.7)		6.12 (2.91–12.9)
2–3	17 (4.9)	90 (46.4)		62.68 (27.2–14.5)
Unknown	111 (32.0)	48 (24.7)		5.44 (2.60–11.4)
Basic activities of daily living, BADL			< 0.001	
No difficulties, 6	167 (48.1)	44 (22.7)		1.00
Difficulties at least in one, ≤ 5	134 (38.6)	125 (64.4)		3.05 (2.00–4.66)
Unknown	46 (13.3)	25 (12.9)		1.61 (0.87–2.97)
Instrumental activities of daily living, IADL			< 0.001	
No difficulties, 8	96 (27.7)	7 (3.6)		1.00
Difficulties in at least one, ≤ 7	205 (59.1)	162 (83.5)		8.90 (3.98–19.9)
Unknown	46 (13.3)	25 (12.9)		5.54 (2.18–14.1)
Physical functioning, TUG Time, Median (IQR)	18.9 (13.3–26.4)	25.0 (19.6–34.4)	< 0.001	
TUG			< 0.001	
Normal, 1–2	142 (40.9)	48 (24.7)		1.00
Moderately abnormal, 3–4	133 (38.3)	82 (42.3)		1.59 (1.03–2.47)
Markedly abnormal, 5	8 (2.3)	14 (17.2)		4.52 (1.75–11.7)
Unknown	64 (18.4)	50 (25.8)		1.76 (1.05–2.96)
Physical functioning, EMS			< 0.001	
≥ 14	258 (74.4)	107 (55.2)		1.00
< 14	44 (12.7)	51 (26.3)		2.37 (1.47–3.81)
Unknown	45 (13.0)	36 (18.6)		1.57 (0.94–2.61)
Change in living arrangements			< 0.001	
Same or less supported	258 (74.4)	113 (58.2)		1.00
More supported	74 (21.3)	76 (39.2)		2.06 (1.38–3.08)
Unknown	15 (4.3)	5 (2.6)		0.76 (0.26–2.14)
Change in mobility level			< 0.001	
Same or improved	247 (71.2)	94 (48.5)		1.00
More impaired	89 (25.6)	94 (48.5)		2.38 (1.51–3.50)
Unknown	10 (2.9)	6 (3.1)		1.45 (0.50–4.17)
Change in nutritional status, MNA-SF			0.031	
Same or better	218 (62.8)	100 (51.5)		1.00
Worse	77 (22.2)	60 (30.9)		1.63 (1.07–2.50)
Unknown	52 (15.0)	34 (17.5)		1.18 (0.70–1.96)

*MMSE* mini-mental state examination, *CDT* clock drawing test, *CDR* clinical dementia rating, *TUG* timed up and go, *IQR* interquartile range, *EMS* elderly mobility scale, *MNA-SF* mini-nutritional assessment, short form, *OR* odds ratio, *CI* confidence interval

A follow-up visit to the geriatric clinic was arranged if the diagnostic criteria of a cognitive impairment were not fulfilled or remained unclear. The researcher physician (RJ) manually extracted diagnoses set up to 2 years post-hip fracture from the electronic patient files retrospectively. For some patients, the diagnostic examinations were still ongoing after the two-year follow-up period. All the diagnoses of cognitive disorders found in the electronic patient files were registered, regardless of the place where they were made. The follow-up time of 2 years was chosen to leave enough time for additional investigations to confirm the diagnosis. Access was granted to scrutinize the electronic patient files of both the hospital and primary health care in the area. Files from the private sector were obtained through the national patient data depository.

The geriatric nurse assessed mobility and living arrangements at the time of the hip fracture and 4-6 months postoperatively. Mobility was categorized as independent or non-independent according to assistance needed. Living arrangements were categorized as living in own home with or without organized home care or living in assisted living accommodation or institution providing 24-h care. Alteration in mobility was either same/improved (better mobility or less supported form of living) or impaired (declined mobility or more supported arrangements). Alteration in nutritional status according to the MNA-SF between baseline and follow-up was categorized as same/better or poorer nutritional status.

Information on mortality was taken from the Population Register Center and electronic patient files.

The Ethics Committee of the local hospital district approved the study design. All participants or their representatives gave informed consent.

### Statistical analysis

The distribution of baseline variables and the domains of the outpatient assessment according to whether receiving or not receiving a new diagnosis of cognitive disorder were described by number of patients with percentages for categorical variables. Continuous but skewed variables were described by medians with interquartile ranges. The statistical difference between groups was tested with Pearson’s Chi square test, Fisher’s exact test for categorical variables and t test or Mann–Whitney test of continuous variables. Patients with diagnostic investigations ongoing at the two-year time point were excluded from the analyses.

Age- and gender-adjusted logistic regression analyses with odds ratios (OR) and 95% confidence intervals (CI) were conducted to examine the associations of each of the baseline variables and outpatient domains with a NDCD. IBM SPSS Statistics version 25.0 for Windows software (SPSS Inc. Chicago, Illinois) was used for statistical analyses. P-values under 0.05 were considered statistically significant.

## Results

**Fig. 1:**
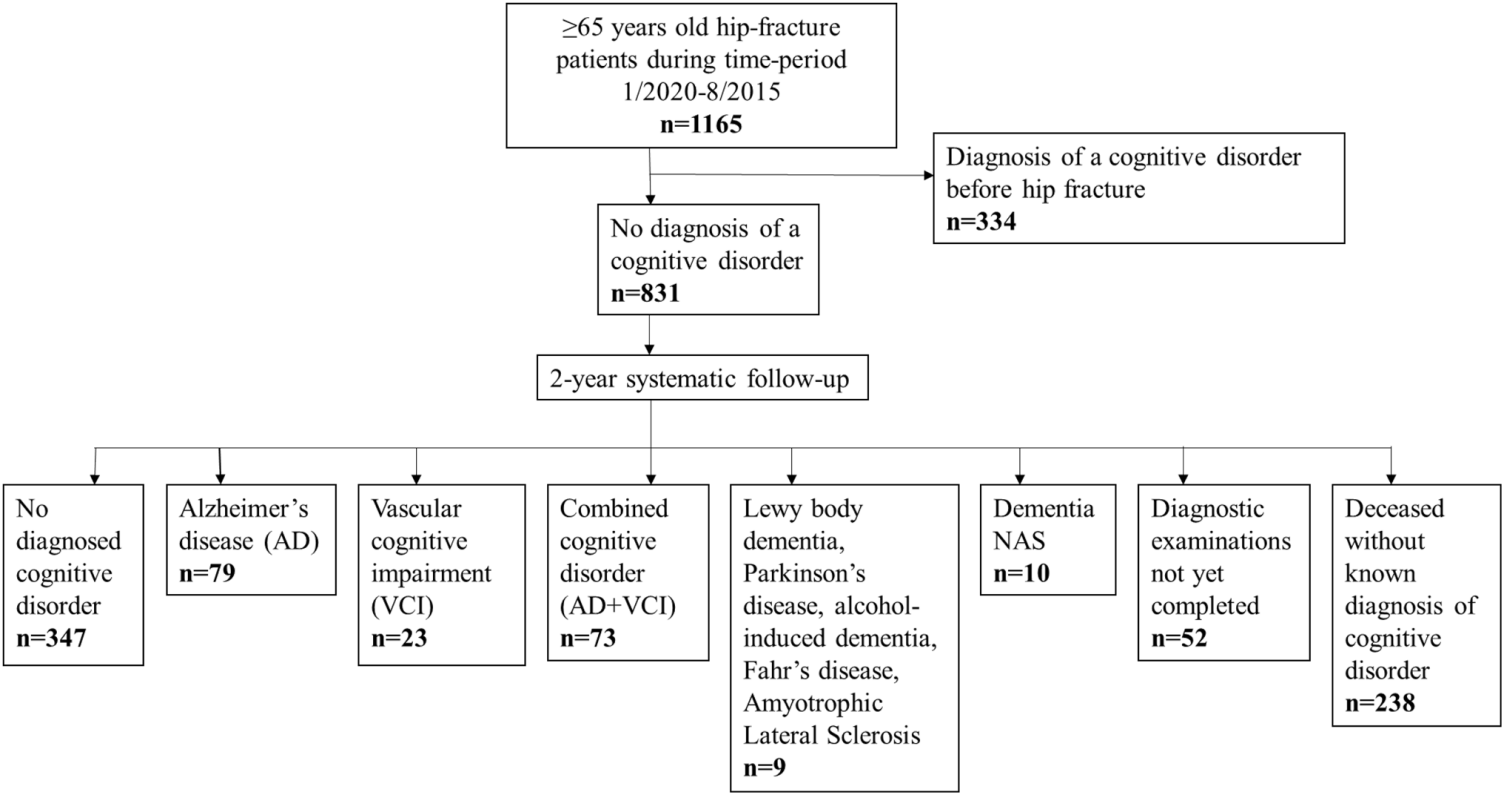
Flow chart of the whole study population leading to new diagnoses of cognitive disorders

A flow chart of the whole study population leading to NDCDs appears in Fig. 1, regardless of the place where the diagnosis was made. In the time period 1,165 patients were treated for their first hip fracture. Approximately one third (*n* = 334, 28.7%) had a diagnosed cognitive disorder at the time of the hip fracture, leaving 831 patients for follow-up. During follow-up 238 (28.6%) patients died while 347 (41.6%) survived without a known diagnosis of cognitive disorder. Diagnostic examinations were ongoing after the follow-up period in 52 (6.3%) cases.

NDCD was documented in 194 patients (23.3%), AD being the most common diagnosis (*n* = 79, 40.7%) followed by mixed cognitive disorder (AD combined with VCI, *n* = 73, 37.6%) and VCI alone (*n* = 23, 11.8%). Lewy body dementia, Parkinson’s disease dementia, alcohol induced dementia and even rarer aetiologies such as dementia with amyotrophic lateral sclerosis and a case of Fahr’s disease were diagnosed in altogether 9 (4.6%) patients. Dementia was deemed undefined in 10 (5.1%) patients (Fig. 1). Of the diagnoses, 170 (87.6%) were made at the geriatric outpatient clinic. Of the remaining 24 diagnoses, 14 were made in the primary health care centres by community geriatricians and seven were made in the private sector by a geriatrician or neurologist. Three diagnoses were made at the department of neurology of the same hospital. The median time from fracture to diagnosis was 8 months (interquartile range from 5 to 14 months).

Of the 831 patients with no pre-fracture diagnosis of a cognitive disorder, 570 (68.6%) attended the geriatric outpatient clinic. Of these, 541 (94.9%) patients receiving or not receiving a diagnosis of cognitive disorder during the two-year follow-up entered the analyses.

Table 1 shows the distribution of baseline characteristics in patients having or not having a NDCD during two-year follow-up and the predictive association of these characteristics with NDCD. Patients were significantly older in the cognitive disorder group. NDCD was more likely in the higher ASA groups and in patients with excessive number of medications. Being at risk of malnutrition according to the MNA-SF was significantly associated with NDCD. Non-independent mobility level and living in more supported circumstances than own home at the time of the hip fracture were associated with cognitive disorder within 2 -year follow-up.

Domains of the post-hip fracture assessment 4–6 months postoperatively according to having or not having NDCD during the 2-year follow-up are shown in Table 2. All cognition assessment methods showed a statistically significant association with NDCDs. Patients with NDCD had more difficulties in activities of daily living. Poor performance on both TUG and EMS was associated with NDCDs. Needing more supported living arrangements and declining mobility were associated with NDCDs. Poorer nutritional status showed a statistically significant association with NDCDs.

## Discussion

This study demonstrated that previously undiagnosed or emerging cognitive disorders are common in older hip fracture patients. Of the baseline factors, higher age and ASA score, non-independent mobility level, not living in own home and being at risk for malnutrition were significantly associated with NDCD. Compared to the pre-fracture situation, patients receiving a new diagnosis of cognitive disorder were more likely to move to a lower mobility level, more supported living arrangements and to have poorer nutritional status than patients without NDCD. Moreover, NDCD was significantly associated with domains indicating disability and impaired physical functioning included in the postoperative CGA.

It is worth noting that a significant proportion (28.6%) of our patients had died without a known diagnosis of a cognitive disorder and may have missed the investigations before dying. Even in the group of patients who survived up to 2 years with no NDCD there may have been some who were out of reach of diagnostic investigations due to e.g. unwillingness or inability to attend the organized follow-up. For these reasons, there appears to be a clear risk of selection bias in our results on NDCDs. We believe that the direction of the bias is more likely towards underreporting than over reporting of NDCDs. Nevertheless, altogether more than 50% of the patients had a pre-fracture diagnosis of cognitive disorder or received such a diagnosis post-fracture. This proportion is in accordance with previously reported prevalence figures of dementia in older hip fracture patients [5, 7]. Interestingly, the distribution of NDCDs corroborates that observed in general older population [24]. AD with or without VCI was the most common diagnosis followed by VCI only. Moreover, cognitive disorders with unusual aetiologies were also observed.

Strikingly, cognitive status in patients with NDCD was in most cases significantly impaired—almost half had moderate dementia according to the MMSE and approximately one tenth had severe dementia. CDT and CDR revealed similar results, suggesting that a cognitive disorder was more likely to have progressed to a moderate or severe stage than merely mild stage at the time of the diagnosis. Furthermore, more frequent difficulties in both BADLs and IADLs compared to the non-NDCD group also suggests a more advanced stage of cognitive disorder at diagnosis. It is possible that acute delirium associated with traumatic injury, hospitalization and operative care may have hastened the development of a gradually exacerbating cognitive disorder [25, 26]. Postoperative delirium has been found to predict development of dementia even in hip fracture patients [26, 27] and has been found to be a risk factor for further cognitive decline in hip fracture patients with pre-existing cognitive impairment [28]. Unfortunately, in our study we were not able to examine the potential effect of delirium on the development of a NDCD. The Confusion Assessment Method (CAM) was included in the original design of data collection but this was not implemented reliably enough and could, therefore, not be included in the analyses. Underdiagnostics of cognitive disorders is still a big global challenge in general older populations [29]. As pointed out in a study by Cherubini and colleagues [30], patients living in long-term care may not have proper access to diagnostic investigations for cognitive disorders. This may also explain the more advanced stage of dementia in some cases in our study. Indeed, patients with a NDCD in our systematic follow-up were more likely to be living in more supported living accommodation at the time of the hip fracture than were those with no NDCD.

It is worth noting that at baseline patients with NDCD were more likely to be at risk of malnutrition as measured by the MNA-SF than were those without. Moreover, patients with NDCD were more likely to have developed poorer nutritional status at follow-up. Weight loss may be one of the first symptoms in Alzheimer’s disease and risk of malnutrition is known to increase as the disease progresses. We have previously reported that poor nutritional status as measured by the MNA-SF was associated with both psycho-cognitive and physical domains of post-hip fracture CGA [31].

One of the major findings of this study was that patients with NDCD had lower scores on both TUG and EMS conducted by physiotherapists. In a study by Friedman and colleagues, lower BMI, cachexia or sarcopenia were associated with cognitively impaired patients, especially in the later stages [32]. According to a recent consensus report, TUG is among the recommended practical assessment tools to predict sarcopenia and the risk of falls [33]. The relationship between cognitive capacity and gait has been investigated, with mounting evidence that cognitive functions are decisive in gait control and alterations in gait relate to cognitive decline [3, 5, 32].

Our findings regarding more supported living arrangements, impaired mobility and poorer nutritional status at follow-up than before the fracture in patients with NDCD are worrying and imply an urgent need for more effective rehabilitation combined with effective nutritional care. Hip fracture patients with cognitive impairments are known to be at risk of malnutrition and need specific attention to nutritional care [31]. In addition, hip fracture patients with mild to moderate dementia have been shown to benefit from multidisciplinary geriatric rehabilitation [34]. In spite of the evidence, unfortunately, this has not been widely implemented in Finland.

The strengths of this study include a large population-based sample and prospective design. All hip fracture patients aged 65 or above are treated by the same protocol regardless of socioeconomic factors, medical history, living arrangements or mobility etc. The same systematic approach was used for each case throughout follow-up. The diagnostic criteria for cognitive disorders at the outpatient clinic were analysed according the current care guideline valid in Finland at the time of the study. Well-known standardized and validated measures were included in the outpatient CGA. Moreover, attendance of eligible patients for outpatient assessment was exceptionally high.

The study also has a number of limitations. First, a major limitation was that the occurrence and prognostic value of delirium could not be included in the analyses. This certainly warrants more attention in future studies. Second, the pre-fracture factors were limited. Moreover, different types of diagnostic procedures for cognitive disorders already diagnosed at the time of the hip fracture were not specified. Third, most domains in the outpatient assessment were included only in the postoperative follow-up visit. For example, pre-fracture cognitive level was unknown. Nevertheless, associations of NDCDs with change in mobility, living arrangements and nutritional status was statistically significant. Finally, the findings presented here reflect the resources and ability of the local health care system to diagnose cognitive disorders and are, therefore, not directly generalizable to other populations. Above all, our study fails to report the true incidence of NDCDs due to a selection bias attributable to the high number of patients who died without a known diagnosis of a cognitive disorder or who were otherwise out of reach of the diagnostic investigations during the two-year follow-up.

## Conclusion

Our study demonstrated that NDCDs were common in older hip fracture patients and had often reached moderate to severe stage before diagnosis. Follow-up assessment must be able to diagnose cognitive disorders and hip fracture patients’ rehabilitation should focus on assessing cognitive status. A comprehensive, systematic post-hip fracture pathway with expertise to diagnose cognitive disorders in the form of a memory clinic seems feasible for the purpose. At population level, earlier diagnosis of cognitive disorders combined with preventive strategies to maintain adequate nutrition and physical functioning as well as to reduce the risk of falls and fractures are warranted.
